# The Multifaceted Roles of Myrrha in the Treatment of Breast Cancer: Potential Therapeutic Targets and Promises

**DOI:** 10.1177/15347354241309659

**Published:** 2024-12-21

**Authors:** Anwar Shams, Abdullah Ahmed Alzahrani, Taghreed A Ayash, Shadi Tamur, Majed AL-Mourgi

**Affiliations:** 1 Department of Pharmacology, College of Medicine, Taif University, Taif, Saudi Arabia; 2 Research Center for Health Sciences, Deanship of Graduate Studies and Scientific Research, Taif University, Taif, Saudi Arabia; 3 High Altitude Research Center, Taif University, Taif, Saudi Arabia; 4 College of Medicine, Taif University, Taif, Saudi Arabia; 5 Department of General Science, Ibnsina International Medical College, Jeddah, Saudi Arabia; 6 Research and Innovation Central lab, Chair of Research and Innovation Central Lab, Ibnsina International Medical College, Jeddah, Saudi Arabia; 7 Department of Pediatric, College of Medicine, Taif University, Taif, Saudi Arabia; 8 Department of Surgery, College of Medicine, Taif University, Taif, Saudi Arabia

**Keywords:** myrrh, bioinformatics, bioactive molecules, genes’ signature, inflammation, breast cancer

## Abstract

**Background:**

Breast cancer is a critical threat to human health, and effective targeted agents showing lower systemic toxicity are still lacking. Therefore, exploring new potent therapeutic candidates with a broader safety margin is warranted. Alternative medicine, which has historically been used in traditional Chinese medicine, has played an increasingly prominent role in this area of research. This study introduces Commiphora myrrha (or myrrh) as a potential therapeutic candidate for treating breast cancer patients. Myrrh bioactive extracts have been used traditionally for decades to treat numerous medical disorders, including cancers, specifically breast cancer. Nonetheless, myrrh’s precise rudimentary mechanisms of action in regulating genes involved in breast cancer evolution and progression remain elusive.

**Purpose:**

Herein, we use a network pharmacology platform to identify the potential genes targeted by myrrh-active molecules in breast cancer.

**Method:**

The identified targets’ expression profiles were determined at the mRNA and protein levels using The Breast Cancer Gene-Expression Miner v5.0 (bcGen-ExMiner v5.0) and The Human Protein Atlas datasets, respectively. A gene signature composed of the specifically designated genes was constructed, and its association with different breast cancer molecular subtypes was investigated through the Gene expression-based Outcome for Breast Cancer (GOBO) online tool. The protein mapping relationship between potential myrrh targets and their partner proteins during breast cancer development was screened and constructed through the STRING and ShinyGO databases. In addition, the Kaplan-Meier plots (KM-plot) prognostic tool was applied to assess the survival rate associated with the expression of the current gene signature in different human cancers, including breast cancer.

**Results:**

Combining the results of network pharmacology with other bioinformatics databases suggests that myrrh’s active components exert anti-cancer effects by regulating genes involved in breast cancer pathogenesis, particularly PTGS2, EGFR, ESR2, MMP2, and JUN. An individual evaluation of the expression profiles of these genes at both mRNA and protein levels reveals that a high expression profile of each gene is associated with breast cancer advancement. Moreover, the GOBO analysis shows an elevated expression profile of the PTGS2/ESR2/EGFR/JUN/MMP2 genes’ signature in the most aggressive breast cancer subtype (Basal) in breast tumor samples and breast cancer cell lines. Furthermore, the STRING protein interaction network and the KEGG analyses indicate that myrrh exerts therapeutic effects on breast cancer by regulating several biological processes such as cell proliferation, cell migration, apoptosis, and various signaling pathways, including TNF, PI3K-Akt, NF-κB, and MAPK. Consistently, breast cancer patients with high expression of this genes’ signature display poor survival outcomes.

**Conclusions:**

The present study is the first attempt to explore the biological involvement of myrrh-targeted genes during breast cancer development. Therefore, suppressing the effects of the intended genes’ signature using myrrh extracts would provide encouraging results in blocking breast cancer tumorigenesis. Thus, our findings provide conclusive evidence and deepen the current understanding of the molecular role of myrrh in the treatment of breast cancer, further supporting its clinical application.

## Graphical abstract


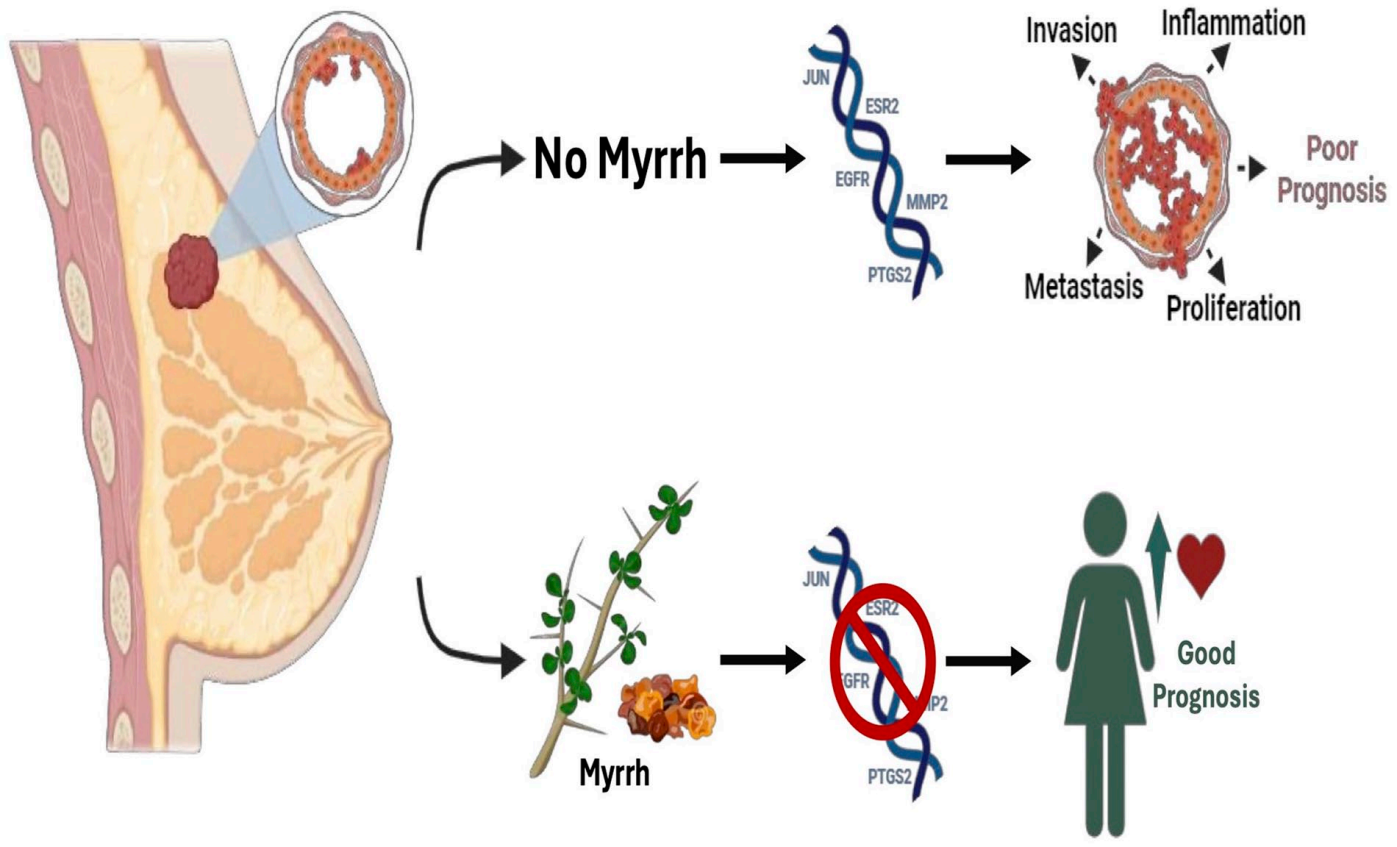


## Background

Breast cancer is a substantial medical concern and represents the most diagnosed cancer among women worldwide.^1^ Despite progress in diagnosing and treating breast cancer, it remains a prevailing cause of cancer death in females globally, with approximately 2.26 million cases reported in 2020.^1-4^ While breast cancer is more prevalent in developed nations, about half of all new cases and an estimated 60% of breast cancer deaths occur in developing countries.^4,5^ Breast cancer is a clinically heterogeneous illness which can be categorized based on the immunohistochemical expression of the hormone receptors, including progesterone receptors (PR), estrogen receptors (ER), and the human epidermal growth factor receptor2 (HER2), into distinct molecular subtypes.^6-8^ This categorization involves Luminal A (ER+/PR+), Luminal B (ER+/PR+/HER2+), HER2-Enriched (ER-/PR-/HER2+), and Triple-Negative (ER-/PR-/HER2-) breast cancer (TNBC) subtypes.^7,9^

Moreover, studies on gene expression have revealed that breast cancer exhibits heterogeneity, which is based on molecular characteristics. However, it remains unclear whether these molecular traits ultimately impact the clinical approach to managing breast cancer.^8,10^ Therefore, understanding the specific subtype of breast cancer is pivotal in clinical practice for determining the most effective therapeutic strategy. Currently, the standard treatment of breast cancer encompasses a combination of approaches depending on the type and stage of the breast cancer, which usually involves surgical resection, radiation therapy, and chemotherapy.^11^ Additionally, hormonal/ endocrine treatment is commonly used in the cases of hormone receptor-positive breast cancer (ER+ and PR+).^11^ Recently, multi-agent combination therapies, which involve the simultaneous use of two or more drugs targeting different oncogenic pathways, have emerged as a promising approach.^5,12,13^ Indeed, combination therapies are often more effective than single-drug therapies and show reduced toxicity levels over prolonged treatment periods.^12^

Historically, alternative medicine is an active research area that elucidated the efficacy of one of the oldest medicinal plants known as Commiphora Myrrha (myrrh) or *Commiphora molmol*, a plant from the family Burseraceae, which is commonly found in Oman, Somalia, Yemen, and Saudi Arabia.^14^ Over the years, it has been acknowledged that the use of myrrh in treating numerous human illnesses is highly safe and effective, as various *in vitro* and *in vivo* studies have extensively described its biologically active constituents and medical properties.^15-26^ In early cultures, myrrh was utilized in Ancient Egypt for mummy preservation, in Chinese medicine for skin infections, and in India for gallbladder and chest disorders.^15,27^ Research has shown that myrrh contains compounds that display anti-cancer effects through various mechanisms, including anti-inflammatory, apoptosis induction, anti-proliferative, and anti-angiogenic effects.^28,29^

Indeed, previous reports suggested that myrrh extracts can be a natural remedy for breast malignancy.^26,30^ Another study found that myrrh oil inhibited the growth of breast tumor cells and induced apoptosis or programed cell cancer death.^31,32^ Furthermore, the *Xihuang* (XH) pill, a natural traditional medicine, has been used effectively in Chinese medicine for the treatment of several cancers, such as gastric cancer, liver cancer, breast cancer, bone cancer, and lymphoma. Along with other components, the *Mo Yao* (*Commiphora myrrha*) constitutes one of the significant ingredients of the XH compositions. The XH pills treat breast neoplasms by suppressing tumor growth, invasion, and angiogenesis and regulating cancer stemness capacity.^18^ Wu et al found that introducing the Z-Guggulsterone (Z-GS), a potent myrrh constituent, to TNBC cells, MDA-MB-468, and BT-549 cells inhibited cellular proliferation, invasion, and migration capacities. The Z-GS also induced breast cancer cell apoptosis and cell cycle arrest at the G2/M phase via regulation of p53/CCNB1/PLK1 signaling to prevent TNBC progression.^33^ Moreover, myrrh extracts (hexane extracts), myrrh essential oils,^25^ or myrrh conjugated with golden nanoparticles^34^ was reported to produce cytotoxic solid effects, using SRB and MTT biological assays, on the human breast cancer cell lines (MCF-7).^25,34^ Nevertheless, additional research is required to delineate myrrh’s precise mechanistic biology, validate the current findings, and determine myrrh’s safety, efficacy, and preventive roles in breast cancer management.

Herein, we used diverse in silico bioinformatics datasets to investigate the potential therapeutic targets of myrrh bioactive compounds in breast cancer treatment. This study is the first attempt to design a gene signature composed of the intended target genes and further dissect the biological engagements of these targets in breast cancer pathogenesis. We also explored the potential molecular mechanisms and signaling pathways by which myrrh bioactive compounds could produce anti-tumorigenic effects in breast cancers by suppressing the intended targets. Finally, we screened the predictive value of our gene signature expression on patients’ outcomes in different cancer types, including breast cancer cases. The current work ultimately provides valuable insights into the therapeutic significance of myrrh as an anti-cancer agent to promote the development of its natural ingredients for use in weakening breast cancer progression.

## The Purpose of the Study

This study explored the possible molecular mechanism(s) and signaling pathways that myrrh bioactive components use to achieve their anti-tumorigenic effects on breast tumors. As a result, we examined the biological interactions between the anticipated targeted genes and various potentially active myrrh components. Our main goals were to demonstrate the targeted genes’ expression profiles at the levels of protein and mRNA in breast cancer samples and to establish a relationship between the genes’ expression profile and various clinicopathological characteristics, as well as patients’ prognosis. We also determined the interacting partner proteins with the genes under investigation and potential pathways involved in the carcinogenesis of breast cancer. Lastly, we clarified how the examined genes are expressed and dysregulated in other forms of cancer in humans. Therefore, we utilized various computational methodological approaches and powerful bioinformatics tools to build up our study. This approach is well renowned^35-37^ and provides comprehensive insight and understanding of the biological role of Myrrh as a traditional natural medicine in breast cancer treatment.

## Methodologies

### Scheme for Screening Active Components of Myrrh and Predicting Targets in Breast Cancer

The active components of myrrh were extracted from the Traditional Chinese Medicine Systems Pharmacology Database and Analysis Platform (TCMSP; https://old.tcmsp-e.com/tcmsp.php).^38^ The active components of myrrh were chosen based on their targeting capacity of different genes in breast cancer. Then, the potential targets of the active compounds in myrrh in this database were examined. The targets were introduced into the UniProt database (https://www.uniprot.org/)^39^ to obtain the official gene names of the candidate targets as the following: Prostaglandin-endoperoxide synthase 2 (PTGS2 or COX-2), Estrogen receptor alpha (ESR1), Estrogen receptor beta (ESR2), Epidermal growth factor receptor (EGFR), Progesterone receptors (PGR), Heat shock protein 90 alpha family class B member 1 (HSP90AB1), Transcription factor AP-1 ( JUN), 72 kDa type IV collagenase (MMP2), and Cathepsin D (CTSD). Next, the Cytoscape (version 3.10.1) software was used to construct the active component-target nodes network and histogram.

### Breast Cancer Gene-Expression Miner

The Breast Cancer Gene-Expression Miner v5.0 (bcGen- ExMiner v5.0; http://bcgenex.ico.unicancer.fr/BC-GEM/GEM-Accueil.php?js=1)^40,41^ is a statistical online mining tool containing published annotated breast cancer transcriptomic data (RNA-seq, n = 5023 and DNA microarrays, n = 11 552). This tool provides the option to evaluate the gene-expression profile, correlation, and predictive values of various genes of interest in breast cancer’s different molecular subtypes. The biological validation of this tool was thoroughly examined and validated for several genes, as stated in the bc-GenExMiner v5.0 website. The correlations of the candidate nine genes (PTGS2, ESR1, ESR2, EGFR, MMP2, JUN, HSP90AB1, PGR, and CTSD) were heat mapped in all breast cancer patients (all subtypes together) using the gene correlation targeted analysis tool (all RNA-seq data) provided by bc-GenExMiner v5.0 (correlation module focused analysis). Then, the positively correlated genes were selected (PTGS2, ESR2, MMP2, EGFR, and JUN), and their relation to the PTGST2 was mapped and dissected further to extract the positive correlation value.

### The Human Protein Atlas

The Human Protein Atlas allowed us to visualize a map of all human protein-coding genes’ expression patterns in various tissues, cells, and organs—both in healthy tissues and throughout the 20 most prevalent cancer types (http://www.proteinatlas.org/).^42^ The data is cataloged using various atlas platform techniques, including transcriptomics, mass spectrometry-based proteomics, antibody-based techniques, and systems biology. We used this database’s pathology/cancer section to picture the expression level of PTGS2/ESR2/EGFR/JUN/and MMP2 proteins in different cancers, especially breast cancer patients’ samples. This section presents data derived from immunohistochemical image analysis, the KM-Plot prognostic data tool, and mRNA and protein expression from 17 distinct kinds of human cancer.

### Data Mining of GOBO

It is a Gene expression-based Outcome for Breast cancer (GOBO) online database (http://co.bmc.lu.se/gobo/)^43,44^ that includes 1881 breast cancer patients. The co-expression profile of PTGS2/ESR2/EGFR/JUN/MMP2 gene signature was examined in all breast tumors (Merged Gene Set) and different breast cancer cell lines (Neve expression- Merged Gene Set). The plotted boxes and whiskers denoted the expression status of the examined gene signature concerning different clinicopathological criteria, including breast cancer molecular subtypes (Hu’s subtypes classification) and the ER/HR (Estrogen Receptor / Hormonal Receptors) status of breast cancer.

### Protein-Protein Interaction Network (STRING)

We used the Search Tool for the Retrieval of Interacting Genes/Proteins database (STRING v10.5; https://string-db.org/)^45^ to construct the PPI network associated with PTGS2/ESR2/EGFR/JUN/and MMP2 proteins using multiple protein search selection in Homo sapiens organism. This tool provides information about the anticipated proteins’ physical and functional interaction. We started by uploading a given list of the proteins as input; STRING can then search for their binding partners and construct a PPI (protein-protein interaction) network, which is a network that shows all the interactions between the inputted proteins and their binding partners according to specifically selected settings. First, we generated the PPI network associated with HUD based on the inputted proteins, obtaining the anticipated proteins and their neighbors. The interactions were extracted from high-throughput lab experiments and previous knowledge in curated databases at a medium confidence level (sources: text-mining, experiments, databases, co-expression, neighborhood, gene fusion, and co-occurrences; score ≥ 0.40). The option of the number of interactors (no more than ten interactions) was selected in our search settings. Next, we used the string analysis system to specify the interactor proteins and identify the ones documented in breast cancer pathogenesis reported by various sources (PubMed, KEGG pathways, and Wiki pathways).

### The Gene Ontology and Kyoto Encyclopedia of Genes and Genomes Pathways Enrichment Analysis

ShinyGO: enrichment tool (http://bioinformatics.sdstate.edu/go/#tab-6415-3, accessed on 28 July 2024),^46^ was accessed to perform the Gene Ontology (GO) analysis and Kyoto Encyclopedia of Genes and Genomes (KEGGs) pathway enrichment. An FDR <0.05 was employed as a cutoff to graph the KEGG signaling pathways and to draw the map of breast cancer’s different dysregulated pathways.

### Assessment of the Effect of the PTGS2/ESR2/EGFR/JUN/MMP2 Gene Signature Expression on Breast Cancer Patients’ Survival Prognosis Using Kaplan-Meier Plots (KM-Plot)

Kaplan-Meier plots were plotted using an online KM plotter database (http://kmplot.com/analysis/).^47^ This tool analyzes the effect of around 54 675 genes (mRNA, miRNA, protein) on the survival outcome in 21 cancer types using 10 293 cancer samples. This analysis is based on gene arrays, RNA-sequence, or next-generation sequencing (for mutation data) extracted from the Affymetrix microarray data in the Gene Expression Omnibus (GEO: http://www.ncbi.nlm.nih.gov/geo/), the European Genome phenome Archive (EGA: https://ega.crg.eu/), and The Cancer Genome Atlas (TCGA: http://cancergenome.nih.gov/) databases. The co-expression of the PTGS2/ESR2/EGFR/JUN/MMP2 genes signature was assessed using several parameters, including Overall Survival (OS), Distant Metastases-Free Survival (DMFS), and Relapse-Free Survival (RFS). The co-expression of these gene signatures in the breast cancer basal subtype (st. Gallen classification) was investigated in a sizable cohort of individuals with breast cancer (~5143 tumors). Also, the prognostic value of the co-expression of the examined gene signature was assessed in other human cancers. The auto-select best cut-off option was used to divide the patient cohort. In all three investigated parameters—OS, RFS, and DMFS—10 years (120 months) is the chosen follow-up criterion. The log-rank P-values (below 0.05 were deemed significant) and the values of each group are shown as the mean ± SD. The hazard ratio (HR) with 95% confidence intervals was calculated automatically by the website tool. For quality assurance, biased arrays were disregarded. The multigene classifier option and the mean expression levels of the examined genes tools offered by the KM plotter were used in this analysis. The Affymetrix IDs identify the examined genes as the following: PTGS2 (204748_at), EGFR (1565483_at), MMP2 (201069_at), JUN (201466_s_at), and ESR2 (211120_x_at).

## Results

### Identification of Myrrh Target Genes in Breast Cancer Using Network Pharmacology-Based Analysis

**Figure 1.**
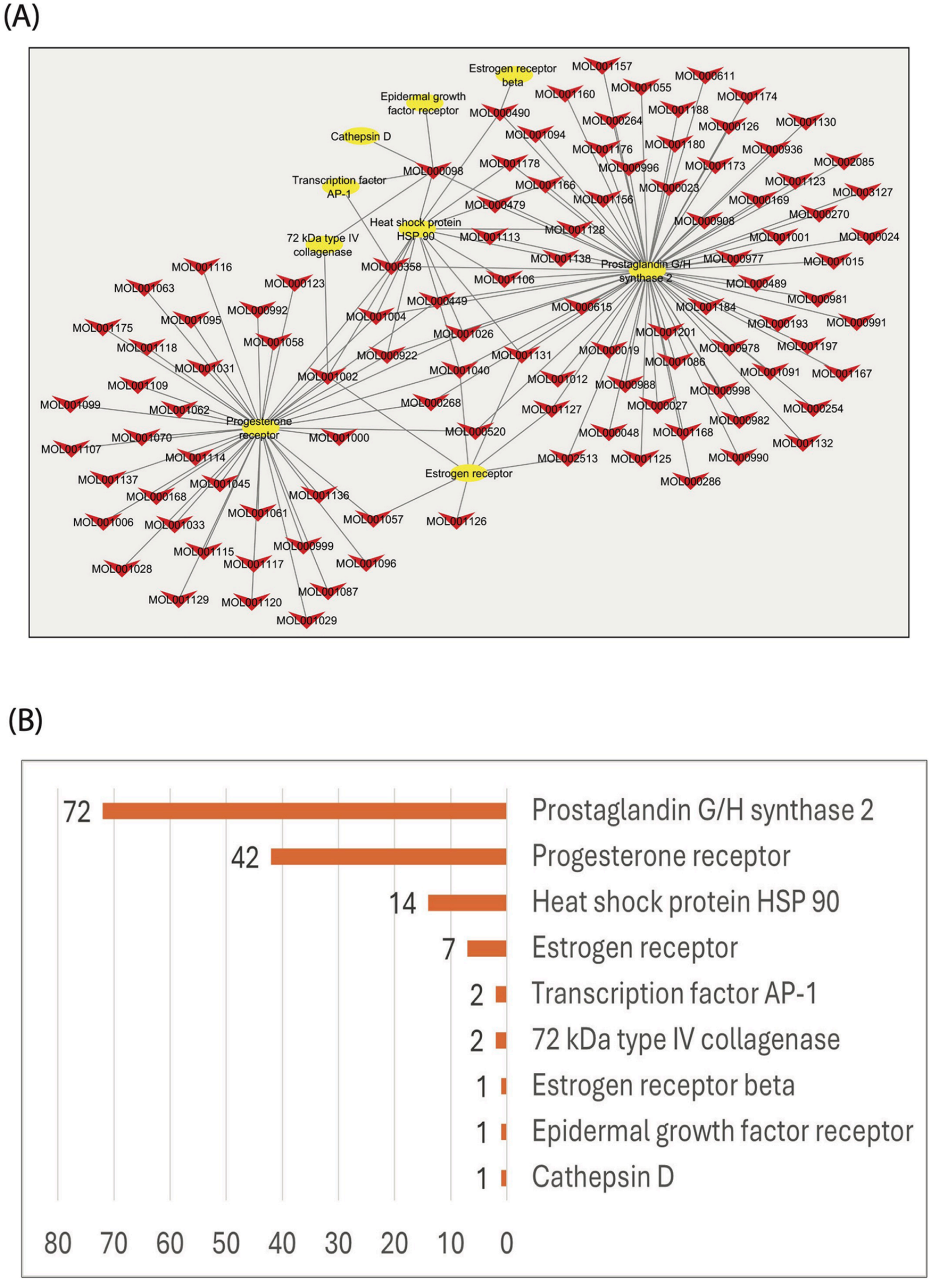
In silico analysis represented the relationship between myrrh molecules composition and the targeted genes in breast cancer using TCMSP (Traditional Chinese Medicine Systems Pharmacology Databases and Analysis Platform): (A); Complex network for the relationship between breast cancer genes (yellow nodes) that are predicted to be targeted by specific myrrh molecules (red nodes), the edges represent the interactions between them. (B); Histogram demonstrated the number of different myrrh molecules which target the predicted breast cancer genes, the abscissa represents the number of nodes for myrrh molecules, and the ordinate is the name of the core target gene.

To investigate the effect of myrrh treatment in breast cancer, we begin with an in-silico dataset exploration of active ingredients in myrrh extracts using the bioinformatics tools that are available online. The active compounds of myrrh were obtained from the TCMSP database.^38^ After eliminating overlapping compounds, as shown in Table 1, 107 bioactive components were determined, and only nine target genes related to breast cancer were retrieved. These genes are PTGS2, EGRF, ESR2, ESR1, PGR, MMP2, JUN, CTSD, and HSP90AB1. The main active myrrh component that was highly effective against BC, according to our analysis, was quercetin (MOL000098). We found that quercetin targeted most of the predicted candidates, including Cathepsin D (CTSD), Epidermal growth factor receptors (EGFR), type IV collagenase (MMP2), Transcription factor AP-1(JUN), Heat shock protein HSP 90 (HSP90AB1), and Prostaglandin G/H synthase 2 (PTGS2), (Table 1). A comprehensive network diagram was constructed to characterize further the interaction between these bioactive myrrh components and their predicted target genes in breast cancer, as demonstrated in Figure 1A. Next, we built a histogram plot (Figure 1B) that presented the high-degree target candidates and their correlation with the number of bioactive myrrh components that affect each predicted gene. Indeed, each target gene can be subjected to the effects of at least one bioactive myrrh component. For instance, CTSD is targeted by the quercetin ingredient, while PTGS2 was predicted to be targeted by 72 bioactive myrrh compounds (the most common targeted gene). Thus, we identified PTGS2 as the key targeted gene. These findings indicate that these highly correlated targets in the network may play an essential therapeutic role in the treatment with myrrh in breast cancer.

**Table 1.** The Effective Bioactive Ingredients of Myrrh Predicted to Target Specific Genes in Breast Cancer.

Mol ID	Molecule name	Target name
MOL000098	quercetin	Cathepsin D
MOL000098	quercetin	Epidermal growth factor receptor
MOL000490	petunidin	Estrogen receptor beta
MOL001002	ellagic acid	72 kDa type IV collagenase
MOL000098	quercetin	72 kDa type IV collagenase
MOL000358	beta-sitosterol	Transcription factor AP-1
MOL000098	quercetin	Transcription factor AP-1
MOL001002	ellagic acid	Estrogen receptor
MOL001040	(2R)-5,7-dihydroxy-2-(4- hydroxyphenyl)chroman-4-one	Estrogen receptor
MOL002513	Xanthorrhizol	Estrogen receptor
MOL001057	2- methyl-5-(5΄ -hydroxy-1΄,5΄ -dimethyl-3΄ -hexenyl)phenol	Estrogen receptor
MOL001126	[(5aS,8aR,9R)-8-oxo-9-(3,4,5- trimethoxyphenyl)-5,5a,6,9- tetrahydroisobenzofurano[6,5- f][1,3]benzodioxol-8a-yl] acetate	Estrogen receptor
MOL001127	erlangerin D	Estrogen receptor
MOL001131	phellamurin_qt	Estrogen receptor
MOL001002	ellagic acid	Heat shock protein HSP 90
MOL001004	pelargonidin	Heat shock protein HSP 90
MOL001026	myrrhanol C	Heat shock protein HSP 90
MOL001040	(2R)-5,7-dihydroxy-2-(4- hydroxyphenyl)chroman-4-one	Heat shock protein HSP 90
MOL001106	ledene	Heat shock protein HSP 90
MOL001128	7-O-methylaloeresin A	Heat shock protein HSP 90
MOL001131	phellamurin_qt	Heat shock protein HSP 90
MOL001138	(3R,20S)-3,20-dihydroxydammar- 24-ene	Heat shock protein HSP 90
MOL001166	4,5-dihydrofuranodiene-6-one	Heat shock protein HSP 90
MOL001178	α-gurjunene	Heat shock protein HSP 90
MOL000358	beta-sitosterol	Heat shock protein HSP 90
MOL000490	petunidin	Heat shock protein HSP 90
MOL000922	(R)-p-Menth-1-en-4-ol	Heat shock protein HSP 90
MOL000098	quercetin	Heat shock protein HSP 90
MOL001000	quercetin-3-O-α-L-rhamnoside	Progesterone receptor
MOL001002	ellagic acid	Progesterone receptor
MOL001004	pelargonidin	Progesterone receptor
MOL001006	poriferasta-7,22E-dien-3beta-ol	Progesterone receptor
MOL001026	myrrhanol C	Progesterone receptor
MOL001028	(8R)-3-oxo-8-hydroxy-polypoda - 13E,17E,21-triene	Progesterone receptor
MOL001029	myrrhanones B	Progesterone receptor
MOL001031	epimansumbinol	Progesterone receptor
MOL001033	diayangambin	Progesterone receptor
MOL001040	(2R)-5,7-dihydroxy-2-(4- hydroxyphenyl)chroman-4-one	Progesterone receptor
MOL001045	(13E,17E,21E)-8-hydroxypolypodo- 13,17,21-trien-3-one	Progesterone receptor
MOL001057	2- methyl-5-(5΄ -hydroxy-1΄,5΄ -dimethyl-3΄ -hexenyl)phenol	Progesterone receptor
MOL001058	picropolygamain	Progesterone receptor
MOL001061	(16S, 20R)-dihydroxydammar-24-en-3-one	Progesterone receptor
MOL001062	15α-hydroxymansumbinone	Progesterone receptor
MOL001063	28-acetoxy-15α-hydroxymansumbinone	Progesterone receptor
MOL001070	3-epi-lupenyl acetate	Progesterone receptor
MOL001087	3-epi-α-amirin	Progesterone receptor
MOL001095	isofouquierone	Progesterone receptor
MOL001096	α-amirone	Progesterone receptor
MOL001099	p-xylene	Progesterone receptor
MOL001107	β-cedrene	Progesterone receptor
MOL001109	β-thujene	Progesterone receptor
MOL001114	2-[(1R)-2,2,3-trimethyl-1-cyclopent-3- enyl]ethanal	Progesterone receptor
MOL001115	cis-Verbenol	Progesterone receptor
MOL001116	pinocarvone	Progesterone receptor
MOL001117	p-mentha-1,5-dien-8-ol	Progesterone receptor
MOL001118	p-mentha-1(7)-dien-8-ol	Progesterone receptor
MOL001120	myrtenal	Progesterone receptor
MOL001129	l-Verbenone	Progesterone receptor
MOL001136	1,2-epoxyfurano-10(15)-germacren-6-one	Progesterone receptor
MOL001137	4,7-dimethoxy-5-methyl-coumarin	Progesterone receptor
MOL001175	Guggulsterone	Progesterone receptor
MOL000123	geraniol	Progesterone receptor
MOL000168	()-2-Carene	Progesterone receptor
MOL000268	(1S,5S)-1-isopropyl-4- methylenebicyclo[3.1.0]hexane	Progesterone receptor
MOL000358	beta-sitosterol	Progesterone receptor
MOL000449	Stigmasterol	Progesterone receptor
MOL000520	alpha-amyrin	Progesterone receptor
MOL000922	(R)-p-Menth-1-en-4-ol	Progesterone receptor
MOL000992	guggulsterol-III	Progesterone receptor
MOL000999	quercetin-3-O-β-D-galactoside	Progesterone receptor
MOL001001	quercetin-3-O-β-D-glucuronide	Prostaglandin G/H synthase 2
MOL001004	pelargonidin	Prostaglandin G/H synthase 2
MOL001012	(5R,8R,9R,10R,13R,14R)-4,4,8,10,14- pentamethyl-1,2,5,6,7,9,11,12,13,15- decahydrocyclopenta[a]phenanthren-3-one	Prostaglandin G/H synthase 2
MOL001015	ZINC00388662	Prostaglandin G/H synthase 2
MOL001026	myrrhanol C	Prostaglandin G/H synthase 2
MOL001040	(2R)-5,7-dihydroxy-2-(4- hydroxyphenyl)chroman-4-one	Prostaglandin G/H synthase 2
MOL001055	5-isopropyl-2-methylbicyclo[3.1.0]hex-2-ene	Prostaglandin G/H synthase 2
MOL002513	Xanthorrhizol	Prostaglandin G/H synthase 2
MOL001086	DMT	Prostaglandin G/H synthase 2
MOL001091	lupeol	Prostaglandin G/H synthase 2
MOL001094	cabraleadiol	Prostaglandin G/H synthase 2
MOL001106	ledene	Prostaglandin G/H synthase 2
MOL001113	α-thujone	Prostaglandin G/H synthase 2
MOL001123	muurolene	Prostaglandin G/H synthase 2
MOL001125	erlangerin B	Prostaglandin G/H synthase 2
MOL001127	erlangerin D	Prostaglandin G/H synthase 2
MOL001128	7-O-methylaloeresin A	Prostaglandin G/H synthase 2
MOL001130	Fellavine	Prostaglandin G/H synthase 2
MOL001131	phellamurin_qt	Prostaglandin G/H synthase 2
MOL001132	longipinene	Prostaglandin G/H synthase 2
MOL001138	(3R,20S)-3,20-dihydroxydammar- 24-ene	Prostaglandin G/H synthase 2
MOL001156	3-methoxyfuranoguaia-9- en-8-one	Prostaglandin G/H synthase 2
MOL001157	(6R,8R,9E)-8-methoxy-3,6,10-trimethyl- 6,7,8,11-tetrahydro-5H-cyclodeca[b]furan-4- one	Prostaglandin G/H synthase 2
MOL001160	2-methoxyfuranodiene	Prostaglandin G/H synthase 2
MOL001166	4,5-dihydrofuranodiene-6-one	Prostaglandin G/H synthase 2
MOL001167	β-selinene	Prostaglandin G/H synthase 2
MOL001168	(1S,2S)-2-isopropenyl-4-isopropylidene-1- methyl-1-vinylcyclohexane	Prostaglandin G/H synthase 2
MOL002085	alpha-Cubebene	Prostaglandin G/H synthase 2
MOL001173	(6S,7R,8E)-7-methoxy-3,6-dimethyl-10- methylidene-5,6,7,11- tetrahydrocyclodeca[b]furan-4-one	Prostaglandin G/H synthase 2
MOL001174	rel-1S,2S-epoxy- 4R-furanogermacr-10(15)- en-6-one	Prostaglandin G/H synthase 2
MOL001176	α-copaene	Prostaglandin G/H synthase 2
MOL001178	α-gurjunene	Prostaglandin G/H synthase 2
MOL001180	γ-muurolene	Prostaglandin G/H synthase 2
MOL003127	Germacrene D	Prostaglandin G/H synthase 2
MOL001184	bicyclogermacrene	Prostaglandin G/H synthase 2
MOL001188	dehydroaromadendrane	Prostaglandin G/H synthase 2
MOL001197	2-hydroxyfuranodiene	Prostaglandin G/H synthase 2
MOL001201	(1R,5R,7S)-4,7-dimethyl-7-(4-methylpent-3- enyl)bicyclo[3.1.1]hept-3-ene	Prostaglandin G/H synthase 2
MOL000126	(-)-nopinene	Prostaglandin G/H synthase 2
MOL000169	alpha-Guaiene	Prostaglandin G/H synthase 2
MOL000019	D-Camphene	Prostaglandin G/H synthase 2
MOL000193	(Z)-caryophyllene	Prostaglandin G/H synthase 2
MOL000023	Hemo-sol	Prostaglandin G/H synthase 2
MOL000024	alpha-humulene	Prostaglandin G/H synthase 2
MOL000254	eugenol	Prostaglandin G/H synthase 2
MOL000264	Tereben	Prostaglandin G/H synthase 2
MOL000268	(1S,5S)-1-isopropyl-4- methylenebicyclo[3.1.0]hexane	Prostaglandin G/H synthase 2
MOL000027	alpha-Curcumene	Prostaglandin G/H synthase 2
MOL000270	CHEBI:7	Prostaglandin G/H synthase 2
MOL000286	β-amyrin acetate	Prostaglandin G/H synthase 2
MOL000358	beta-sitosterol	Prostaglandin G/H synthase 2
MOL000449	Stigmasterol	Prostaglandin G/H synthase 2
MOL000479	Farnesene	Prostaglandin G/H synthase 2
MOL000048	(5E,9Z)-3,6,10-trimethyl-4,7,8,11- tetrahydrocyclodeca[b]furan	Prostaglandin G/H synthase 2
MOL000489	(1S,4aR,8aR)-1-isopropyl-7-methyl-4- methylene-2,3,4a,5,6,8a-hexahydro-1H- naphthalene	Prostaglandin G/H synthase 2
MOL000490	petunidin	Prostaglandin G/H synthase 2
MOL000520	alpha-amyrin	Prostaglandin G/H synthase 2
MOL000611	beta-Bourbonene	Prostaglandin G/H synthase 2
MOL000615	delta-amorphene	Prostaglandin G/H synthase 2
MOL000908	beta-elemene	Prostaglandin G/H synthase 2
MOL000922	(R)-p-Menth-1-en-4-ol	Prostaglandin G/H synthase 2
MOL000936	Germacrene B	Prostaglandin G/H synthase 2
MOL000977	furanogermacra-1E,10(15)-dien-6-one	Prostaglandin G/H synthase 2
MOL000978	furanoeudesma- 1,4-diene-6-one	Prostaglandin G/H synthase 2
MOL000098	quercetin	Prostaglandin G/H synthase 2
MOL000981	camphorene	Prostaglandin G/H synthase 2
MOL000982	isofuranogermacrene	Prostaglandin G/H synthase 2
MOL000988	4,17(20)-(cis)-pregnadiene-3,16-dione	Prostaglandin G/H synthase 2
MOL000990	guggulsterol-II	Prostaglandin G/H synthase 2
MOL000991	cinnamaldehyde	Prostaglandin G/H synthase 2
MOL000996	Guggulsterol IV	Prostaglandin G/H synthase 2
MOL000998	2-(3,4-dihydroxyphenyl)-5,7-dihydroxy-3- [(2R,3R,4S,5S)-3,4,5- trihydroxytetrahydropyran-2-yl]oxy- chromone	Prostaglandin G/H synthase 2

### Myrrh-Predicted Targets Showed Various Gene Expression Levels in Correlation With Different Breast Cancer Molecular Subtypes

**Figure 2.**
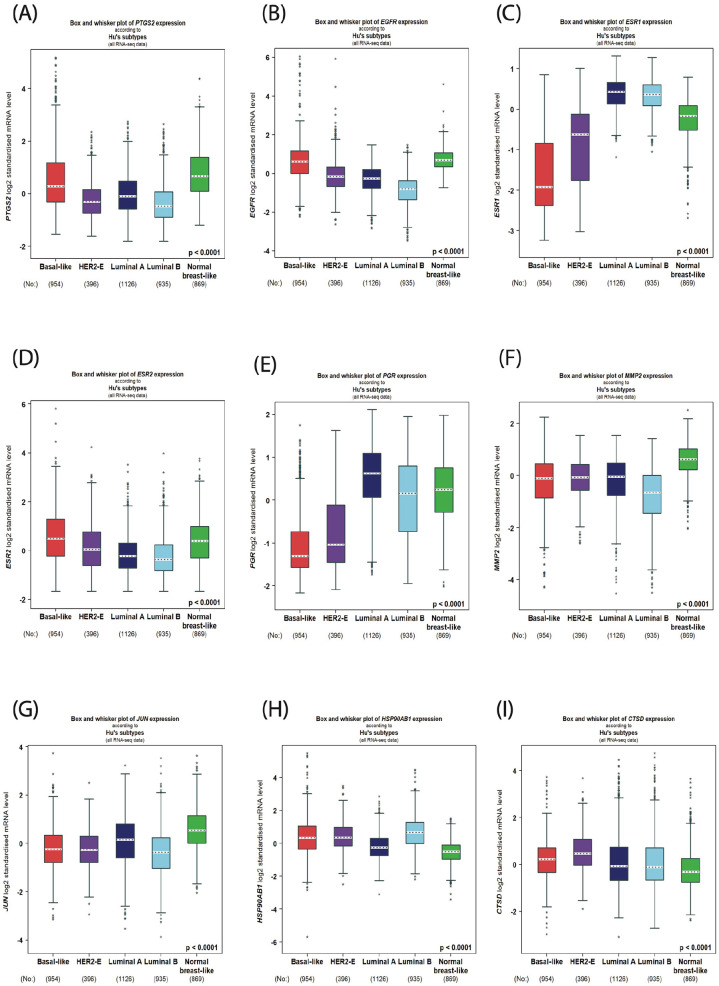
Correlation of the predicted targeted genes by myrrh compositions with different breast cancer molecular subtypes (A-I); Box plot of the targeted genes (PTGS2, EGFR, ESR1, ESR2, PGR, MMP2, JUN, HSP90AB1, and CTSD) expression profile in different breast cancer subtypes according to Hu’s classification using Breast Cancer Gene-Expression Miner v5.0. The number of tumor samples is indicated below each subtype.

**Figure 3.**
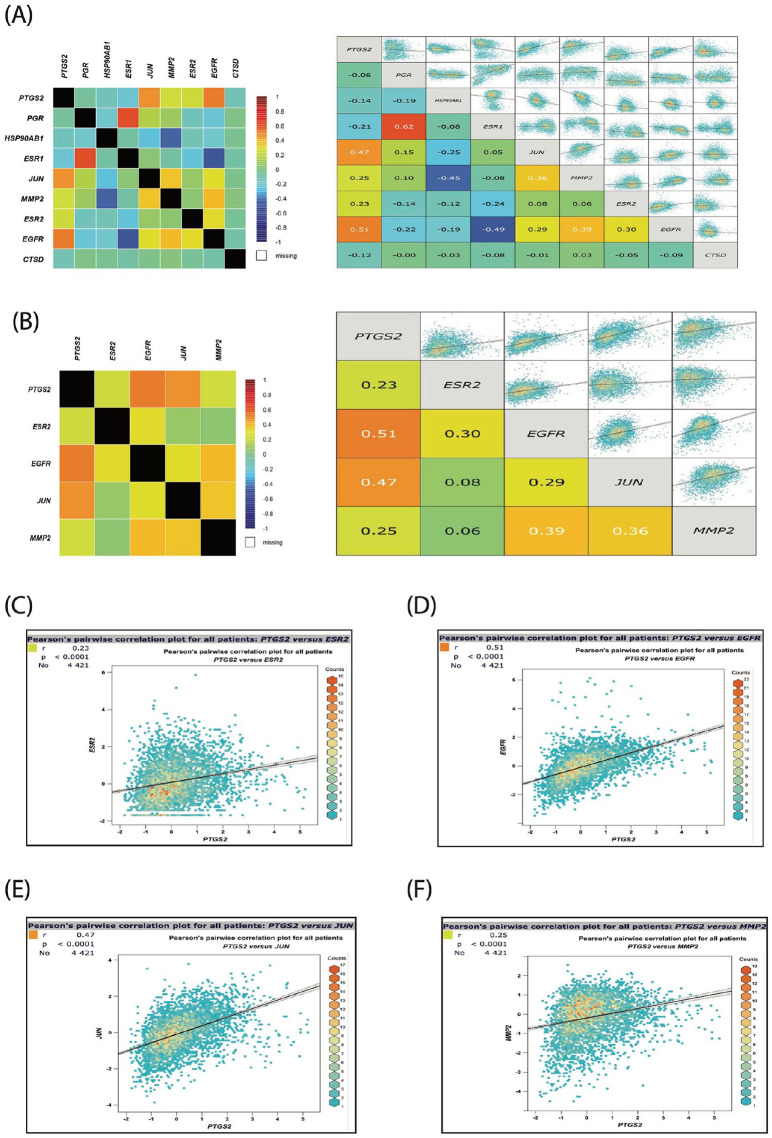
Heat-maps demonstrated different types of expression correlations between the targeted genes and the PTGS2 in breast cancer patients (all subtypes): (A) PTGS2 – Genes correlation maps showing different degrees of correlation strength (left & right panels). These maps presented the correlations between PTGS2 expression and the other eight targets (ESR1, ESR2, PGR, EGFR, JUN, MMP2, HSP90AB1, and CTSD) expression. (B); PTGS2 – Genes correlation map showing different degrees of only positive correlation strength (left & right panels). These maps presented the correlations between PTGS2 expression and the expression of the four selected targets (ESR2, EGFR, JUN, and MMP2). (C-F); Pearson’s pairwise correlation dissecting maps show variable degree of positivity correlated expression strength between PTGS2 and ESR2 (*r* = .23, *P* < .0001), PTGS2 and EGFR (*r* = .51, *P* < .0001), PTGS2 and JUN (*r* = .47, *P* < .0001), and PTGS2 and MMP2 (*r* = .25, *P* < .0001). Breast Cancer Gene-Expression Miner v5.0 plotted these maps.

Our next aim was to dissect the biological engagements of these nine predicted targeted genes by different myrrh-active components in breast cancer pathogenesis. Therefore, we conducted a comprehensive analysis using a publically available database of The Breast Cancer Gene-Expression Miner v5.0 (bcGenExMiner v5.0)^40^ to examine the gene expression level of these candidates in different breast cancer intrinsic molecular subtypes. Our results (Figure 2A-I) showed that these genes exhibited different expression profiles among distinctive breast cancer molecular subtypes, suggesting various contributions of each gene in breast cancer tumorigenesis. For example, the mRNA expression of both PTGS2 and EGFR were the highest in the basal subtype, and conversely, their mRNA levels revealed the lowest expression in the Luminals (A & B) subtypes (Figure 2A and B). On the other hand, the mRNA expression of ESR1 and PGR was the highest in the Luminals (A & B) subtypes and showed the lowest expression level in the basal subtype (Figure 2C and E). To study these genes accurately, we designated the genes with a relatively common mRNA expression profile based on the highest level. These include PTGS2, EGFR, ESR2, MMP2, JUN, and HSP90AB1, which all revealed the highest mRNA expression level in the basal breast cancer subtype, thus proposing a potential similar engagement of these genes in the pathogenesis of the basal subtype. To further validate our assortment of these genes, we ran another analysis to map the correlation between PTGS2 and the other eight candidates in a large cohort of 5696 breast cancer patients’ samples using the same bcGenExMiner v5.0 platform. The selection of PTGS2 as a critical gene for comparison is based on our previous result (Figure1B), which showed that PTGS2 is the top targeted gene by myrrh compounds, indicating an essential role played by PTGS2 in breast cancer tumorigenesis. Therefore, our next drive was to investigate the genes that show parallel association with PTGS2. As shown in Figure 3A, different correlations between PTGS2 expression and the expression of the other eight genes were obtained and classified as equivalent, negative, or positive associations with various degrees of strength. Interestingly, the highest Pearson’s pairwise positive correlation with the PTGS2 gene was perceived with the following genes: EGFR (.51), JUN (.47), MMP2 (.25), and ESR2 (.23). These results encourage us to continue our analysis of these five genes and map/dissect their correlation in all breast cancer patients’ samples. As demonstrated in Figure 3B to F, a significant (*P* < .0001) positive Pearson’s pairwise correlation with different strength intensities was observed between PTGS2 and the other four genes in the heatmaps representing all breast cancer patients. Together, these findings indicate a subsequent involvement of these five genes in breast cancer development and progression. Yet, the exact mechanistic role provided by each gene or as a group needs further exploration.

### Myrrh-Predicted Targets Exhibited Prominent Protein Expression Levels in Human Breast Cancer Tissues

**Figure 4.**
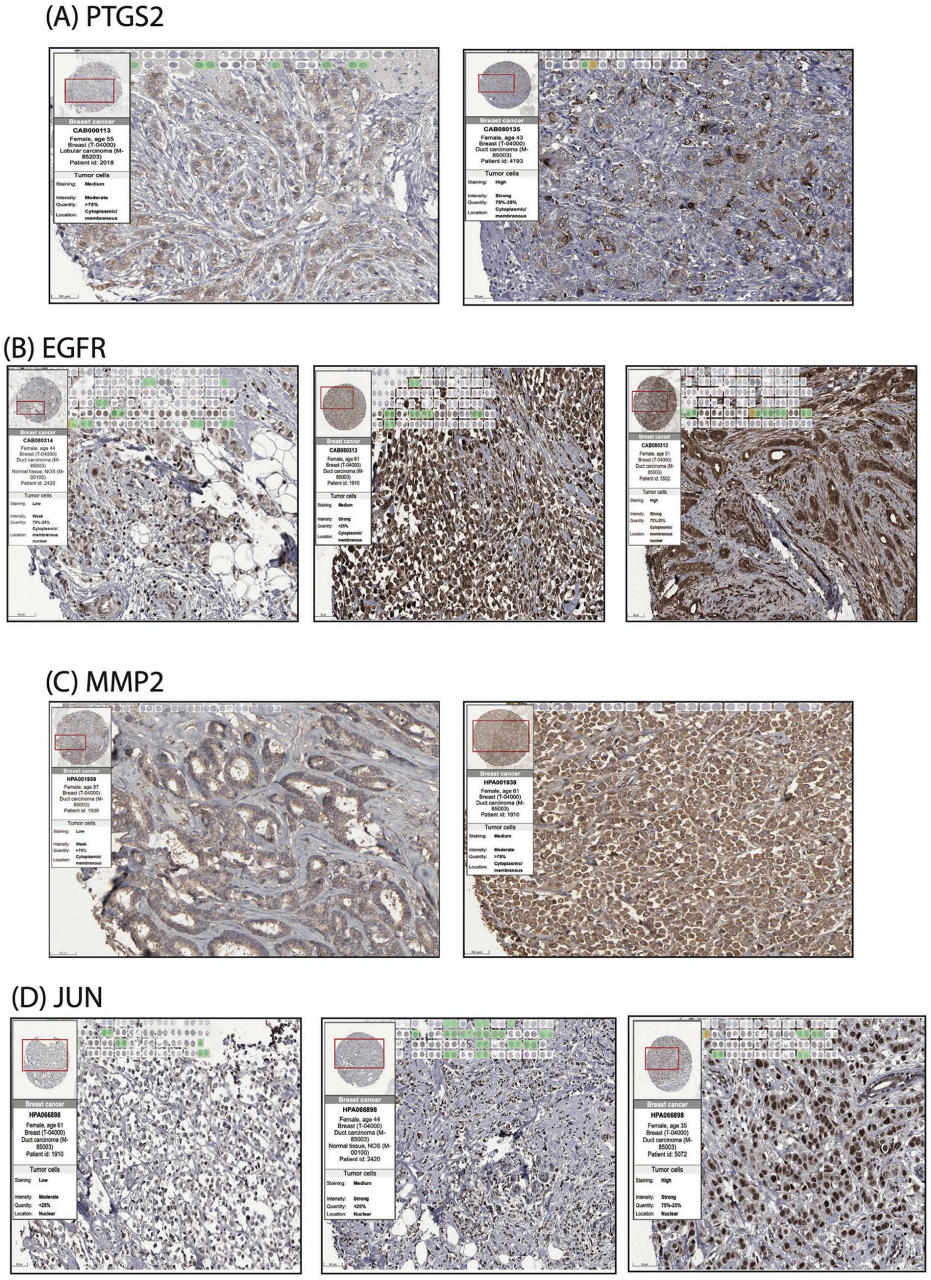
Visualization of PTGS2, EGFR, MMP2, and JUN protein levels profiles in breast cancer patients: (A) Immunohistochemical analysis of PTGS2 expression in 19 breast cancer patients’ samples using CAB000113-ThermoFisher Scientific antibody against PTGS2 protein, (Cytoplasmic/membranous staining: left panel; medium, right panel; high). (B) Immunohistochemical analysis of EGFR expression in 24 breast cancer patients’ samples using CAB080313-NCI-CPTAC antibody against EGFR protein (Cytoplasmic/membranous staining: left panel; low, middle panel; medium, right panel; high). (C) Immunohistochemical analysis of MMP2 expression in 22 breast cancer patients’ samples using HPA001939-Sigma-Aldrich antibody against MMP2 protein (Cytoplasmic/membranous staining: left panel; low, right panel; medium). (D) Immunohistochemical analysis of JUN expression in 23 breast cancer patients’ samples using HPA066898-Sigma-Aldrich antibody against JUN protein (Nuclear staining: left panel; low, middle panel; medium, right panel; high). All images obtained from THE HUMAN PROTEIN ATLAS online tool have a scale bar of 50 mm.

To study the role of the five assorted genes, PTGS2/ESR2/EGFR/JUN/and MMP2, in breast cancer tumorigenesis, we explored the expression profile of these genes individually in breast cancer tissues at the protein level through THE HUMAN PROTEIN ATLAS online tool.^42^ This database provides information about the expression level of the protein-coding genes from 20 different types of human cancers, including breast cancer, with different histopathological classifications, using the analysis of Immunohistochemistry (IHC). Our search demonstrated that the PTGS2 protein levels in breast cancer patients’ samples (Figure 4A, left & right panels) revealed positive staining (medium to high intensity) in 70% of the examined tissues. Furthermore, the IHC analysis of EGFR protein levels (Figure 4B, left, middle, & right panels) presented positive staining of all examined breast cancer samples, for which 50% showed high intensity (right panel), 42% with medium intensity (middle panel), and only 8% showed low intensity (left panel). Similar data was obtained for JUN and MMP2 proteins (Figure 4C and D), which both displayed positive staining for all tested patients’ samples, which mainly expressed medium intensity staining by 58% and 75%, respectively. Indeed, the strength/intensity of staining was reported to be associated with the aggressivity of cancer and the tumor size.^48,49^ Collectively, these data indicate that the examined genes are upregulated during breast cancer evolution, thus supporting a potential oncogenic role delivered by these genes in breast cancer patients.

### PTGS2/ESR2/EGFR/JUN/MMP2 Genes Signature Demonstrated a Distinguishing Expression Profile in Association With Different Breast Cancer Molecular Subtypes

**Figure 5.**
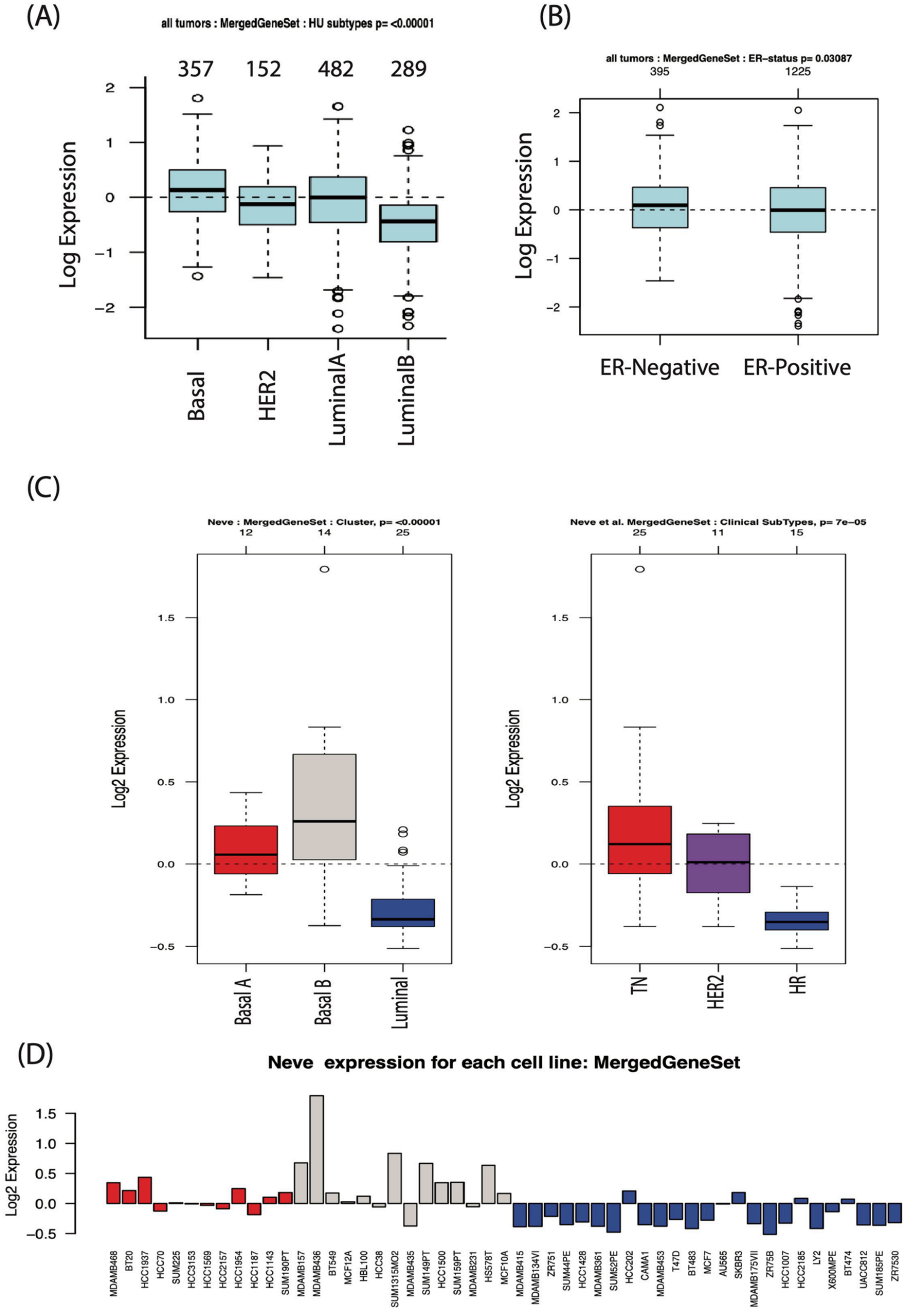
Expression profiles PTGS2/ESR2/EGFR/JUN/MMP2 genes’ signature in correlation with breast cancer molecular subtypes and hormonal receptors status in patients’ tumor samples and cell lines: (A) PTGS2/ESR2/EGFR/JUN/MMP2 genes signature expression level according to breast cancer molecular subtypes, tumor samples, basal (n = 357), HER2 (n = 152), Luminal A (n = 482), and Luminal B (n = 289). (B) PTGS2/ESR2/EGFR/JUN/MMP2 genes signature expression level according to ER status, tumor samples, ER-negative (n = 395) and ER-positive (n = 1225). (C and D) PTGS2/ESR2/EGFR/JUN/MMP2 genes signature expression level in different breast cancer cell lines, Basal A (n = 12), Basal B (n = 14), Luminal (n = 25), TN (n = 25), HER2 (n = 11), and HR (n = 15). Source of data: Gene expression-based Outcome for Breast cancer (GOBO) online database. TN; triple negative, HR; hormonal receptors.

To further dissect the biological contributions of the selected PTGS2/ESR2/EGFR/JUN/and MMP2 genes in breast cancer development, we studied their correlation, as a group, to different breast cancer molecular subtypes and ER / HR status. For this purpose, we used the Gene expression-based Outcome for Breast Cancer Online (GOBO) dataset that contains data about 1881 breast cancer patients^43,44^ to produce a gene set composed of PTGS2/ESR2/EGFR/JUN/ MMP2 genes’ signature. As shown in Figure 5A and B, our results disclosed that higher expression of PTGS2/ESR2/EGFR/JUN/MMP2 genes’ signature associated with the most aggressive breast cancer subtypes donating by basal breast cancer (*P* < .00001) and the ER-negative breast tumors ( *P* < .00001). On the other hand, lower co-expression of this genes’ signature was found in the least aggressive breast cancer subtype involving Luminals (A & B; Figure 5A, *P* < .00001) and ER-positive tumors (Figure 5B, *P* < .00001). Similar results were obtained when examining the co-expression of PTGS2/ESR2/EGFR/JUN/and MMP2 genes’ signature in breast cancer cell lines representative of different molecular subtypes. As can be seen in Figure 5C and D, breast cancer cell lines demonstrative of the most aggressive subtypes, the Basal (A & B, red and gray boxes) and TNBC subtypes (red boxes) showed the highest expression profile of this genes’ signature. In contrast, the Luminals (A & B)/HR subtypes, which unveil the least aggressive phenotype (blue boxes), revealed the lowest expression intensity. Accordingly, these data should clarify the potential oncogenic role delivered by the simultaneous expression of the current intended genes in patients with breast cancer. This expression is associated with advanced breast cancer subtypes and a higher degree of malignancy, both of which suggest the deprived prognosis of these patients. Thus, suppression/targeting of these gene expressions by the introduction of myrrh treatment might provide a promising tool in impeding breast cancer progression.

### PTGS2/ESR2/EGFR/JUN/and MMP2 Genes’ Signature Revealed a Network of Interaction With Partner Proteins Involved in Breast Cancer Tumorigenesis

**Figure 6.**
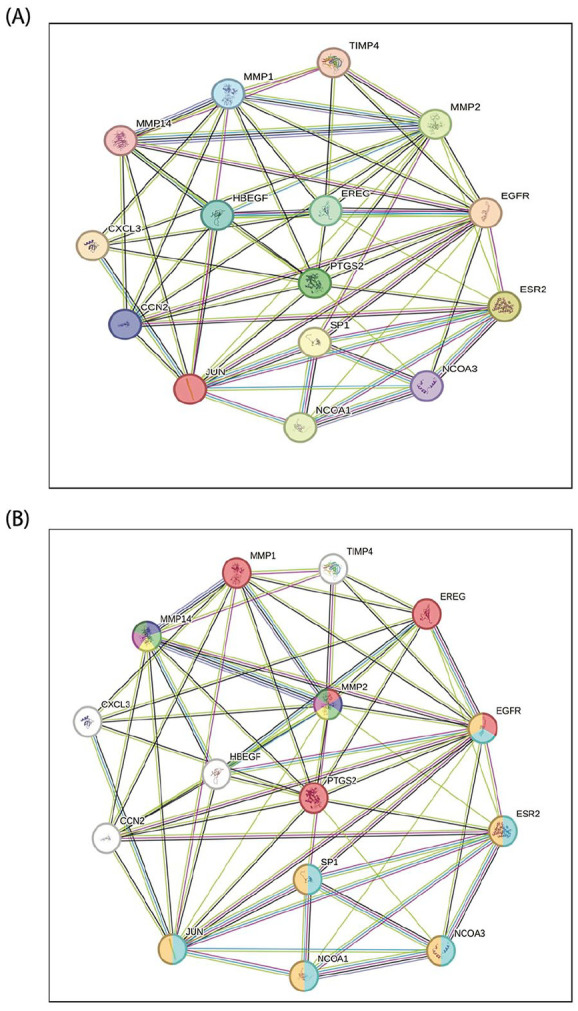
Protein-Protein Interaction network: (A) Diagram shows the network of the PTGS2/ESR2/EGFR/JUN/MMP2 genes’ signature interaction pattern with partner proteins. (B) The diagram shows the network of the PTGS2/ESR2/EGFR/JUN/MMP2 genes’ signature interaction pattern with partner proteins, which are documented to be involved in breast cancer pathogenesis (the colorful nodes). Circular nodes represent the targets, and the edges represent the interaction. All diagrams were generated using the STRING online tool.

It is vital to identify the likely biological processes and cellular functions by which PTGS2/ESR2/EGFR/JUN/MMP2 genes’ signature exhibited their tumorigenic effect in breast cancer. Thus, we scrutinized the interaction between these genes’ signature and other proteins that might also be involved in the processes of breast carcinogenesis. For this objective, we used the Search Tool for the Retrieval of Interacting Genes/Proteins database (STRING v10.5)^45^ to construct the protein-protein interaction (PPI) network associated with these genes’ signature proteins. As shown in Figure 6A, about ten anticipated partners of these genes’ signatures were retrieved in the network at the protein level and showed numerous involvements in regulating many biological processes and cellular functions. Several protein interactors have been identified to be involved in extracellular matrix organization, cell growth, proliferation, differentiation, cellular responses to stimuli, and regulation of cellular processes, including MMP2, MMP1, and MMP14, in addition to the regulation of ERK1/2 pathways such as CCN2. Other interactor proteins were found to be engaged in the regulation of the cellular developmental, metabolic process, and molecular functions (TIMP4 and CXCL3), signal transduction, endocrine resistance and estrogen signaling pathway, and carcinogenesis (NOCA1, NOCA3, SP1, ESR2, and JUN), and the regulation of inflammatory pathways (PTGS2). Additionally, our dissecting of this protein interactions network found that EREG, EGFR, and HBEGF strongly contribute to the regulation of epidermal growth factor receptors activity, cellular proliferation and differentiation, angiogenesis and migration, positive gene expression, and different types of cancer signaling pathways. To isolate the interactors identified to participate in breast cancer pathogenesis, we used specific settings provided by the STRING platform. As shown in Figure 6B, our search resulted in the list of proteins. These colored nodes were documented by different resources (PubMed, KEGG pathways, and Wiki pathways) to participate in breast cancer pathogenesis, including MMP1, MMP2, MMP14, EGFR, EREG, PTGS2, ESR2, SP1, JUN, NOCA1, and NOCA3. The current analysis further certifies the decisive engrossment of PTGS2/ESR2/EGFR/JUN/MMP2 genes’ signature and some interactor proteins in promoting breast cancer development.

### Enrichment Analysis of the Related PTGS2/ESR2/EGFR/JUN/and MMP2 Genes’ Signature Displayed Involvement in Breast Cancer Signalling Pathways

**Figure 7.**
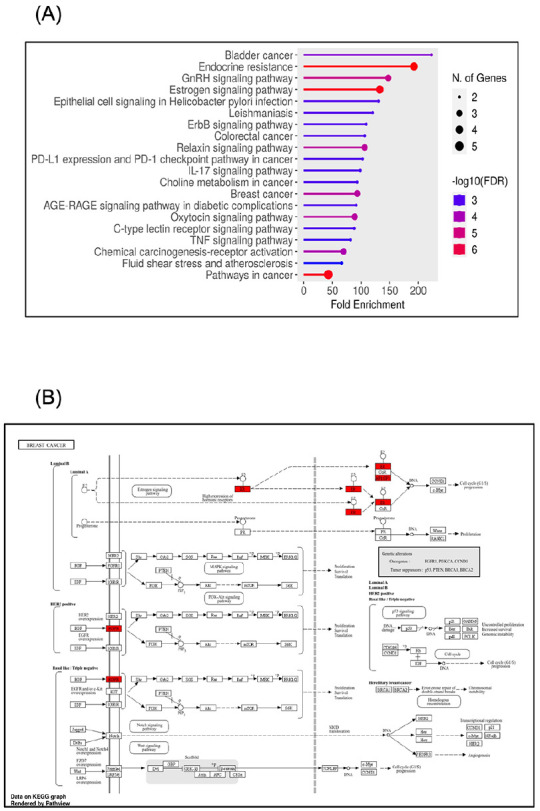
Top 20 significantly enriched Gene Ontology (GO) analyses (sorted by Fold Enrichment): (A) Diagram shows the contributions of the PTGS2/ESR2/EGFR/JUN/MMP2 genes’ signature in various KEGG pathways. (B) Signaling pathways involved in the pathology of different breast cancer molecular subtypes. All diagrams were generated using the ShinyGO online tool.

Numerous signaling pathways, such as the p53, IL-17, PTEN, and PI3K-Akt pathways,^50^ have been altered during breast cancer evolution. Therefore, our next aim was to acknowledge the potential signaling pathways associated with the actions of myrrh bioactive ingredients, specifically quercetin (presented in Table 1) on breast cancer. The KEGG pathway enrichment analysis of the targeted genes’ signature was established (FDR < 0.05) by applying the ShinyGO analysis tool for KEGG and GO enrichment analyses (http://bioinformatics.sdstate.edu/go/). As shown in Table 2 and Figure 7, the KEGG pathway results showed about 20 signaling pathways that were enriched and ranked by the fold enrichment values. Examples of these pathways include bladder cancer, endocrine resistance, GnRH signaling, estrogen signaling, ErbB signaling, colorectal cancer, PD-L1 and PD-1 checkpoints, IL-17 signaling, choline metabolism in cancer, breast cancer, TNF signaling, chemical carcinogenesis, and pathways in cancer (Figure 7A). From this we found our interesting pathways in breast cancer encompassing: Path:hsa01522 Endocrine resistance, Path:hsa04915 Estrogen signaling pathway, and Path:hsa05224 Breast cancer (Table 2). Regarding breast cancer development, we found that our genes’ signature components contribute to the pathogenesis of different breast cancer molecular subtypes. As described in Figure 7B, EGFR plays an essential role in the tumorigenesis of HER2+ and basal/TNBC subtypes through participation in PI3K-Akt and MAPK signaling pathways. At the same time, we showed the engagement of ER and AP-1 (JUN) in the breast cancer luminal subtypes through modulation of the estrogen signaling pathways. Collectively, these findings provide valid evidence supporting the contributions of our genes’ signature in the pathology of breast cancer; hence, targeting these participated genes by myrrh bioactive components would suppress the evolution and progression of breast cancer.

**Table 2.** The KEGG Pathways Enrichment Analysis of the Related Genes’ Signature.

Enrichment FDR	nGenes	Pathway genes	Fold enrichment	Pathway	Genes
0.00035383	2	41	223.229268	Path:hsa05219 Bladder cancer	EGFR MMP2
1.42E-07	4	95	192.682105	Path:hsa01522 Endocrine resistance	EGFR ESR2 JUN MMP2
1.65E-05	3	93	147.619355	Path:hsa04912 GnRH signaling pathway	EGFR JUN MMP2
2.23E-07	4	138	132.643478	Path:hsa04915 Estrogen signaling pathway	EGFR ESR2 JUN MMP2
0.00093548	2	70	130.748571	Path:hsa05120 Epithelial cell signaling in Helicobacter pylori infection	EGFR JUN
0.00100309	2	76	120.426316	Path:hsa05140 Leishmaniasis	JUN PTGS2
0.0010817	2	84	108.957143	Path:hsa04012 ErbB signaling pathway	EGFR JUN
0.0010817	2	86	106.423256	Path:hsa05210 Colorectal cancer	EGFR JUN
3.54E-05	3	129	106.423256	Path:hsa04926 Relaxin signaling pathway	EGFR JUN MMP2
0.0010817	2	89	102.835955	Path:hsa05235 PD-L1 expression and PD-1 checkpoint pathway in cancer	EGFR JUN
0.00110253	2	93	98.4129032	Path:hsa04657 IL-17 signaling pathway	JUN PTGS2
0.00112494	2	98	93.3918367	Path:hsa05231 Choline metabolism in cancer	EGFR JUN
4.31E-05	3	147	93.3918367	Path:hsa05224 Breast cancer	EGFR ESR2 JUN
0.00112494	2	100	91.524	Path:hsa04933 AGE- RAGE signaling pathway in diabetic complications	JUN MMP2
4.31E-05	3	154	89.1467532	Path:hsa04921 Oxytocin signaling pathway	EGFR JUN PTGS2
0.00114919	2	104	88.0038462	Path:hsa04625 C-type lectin receptor signaling pathway	JUN PTGS2
0.00126263	2	112	81.7178571	Path:hsa04668 TNF signaling pathway	JUN PTGS2
7.91E-05	3	197	69.6883249	Path:hsa05207 Chemical carcinogenesis-receptor activation	EGFR ESR2 JUN
0.00181998	2	138	66.3217391	Path:hsa05418 Fluid shear stress and atherosclerosis	JUN MMP2
2.23E-07	5	530	43.1716981	Path:hsa05200 Pathways in cancer	EGFR ESR2 JUN MMP2 PTGS2

### Higher Signature Expression Levels of the PTGS2/ESR2/EGFR/JUN/and MMP2 Genes Are Associated With Poor Patient Outcomes in Breast Cancer

**Figure 8.**
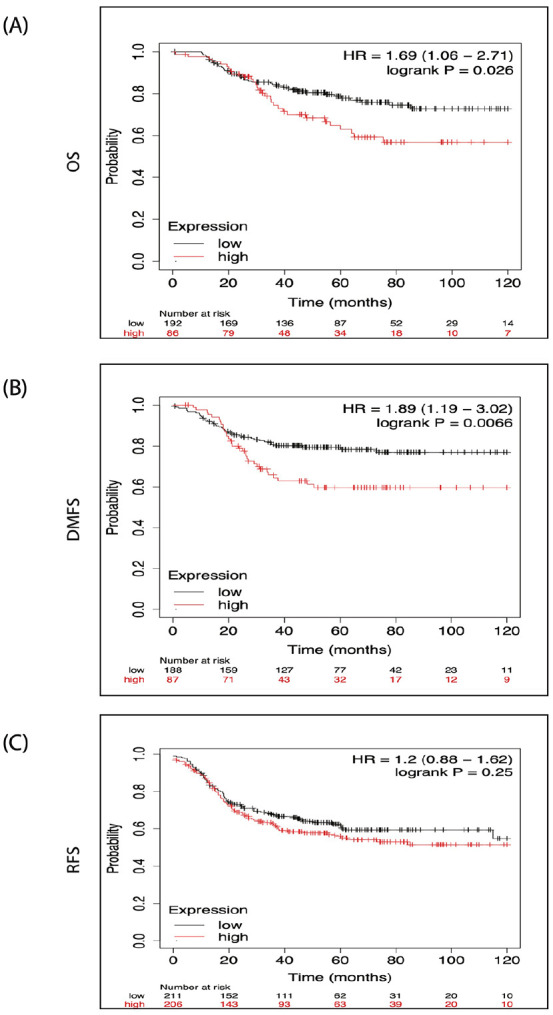
The Kaplan Meier Plotter (KM-Plotter) shows the survival curves denoted by PTGS2/ESR2/EGFR/JUN/and MMP2 genes’ signature expression in breast cancer patients: (A and B) High expression of PTGS2/ESR2/EGFR/JUN/and MMP2 genes’ signature is associated significantly with shorter overall survival (OS), *P* = .026, and distant metastases free survival (DMFS), *P* = .0066, in breast cancer patients – Basal subtype. (C) There is no significant (*P* = .25) correlation between high expression of PTGS2/ESR2/EGFR/JUN/and MMP2 genes’ signature and relapse-free survival (RFS). All curves were obtained using the KM-Plotter analysis prognostic analysis database.

The above results demonstrated the ultimate engagement of PTGS2/ESR2/EGFR/JUN/MMP2 genes’ signature in breast cancer development and progression. To frame the picture, we evaluated the prognostic significance of these genes’ signature expression in breast cancer patient outcomes. For this determination, we used the Kaplan-Meier (KM) plotter. This gene profiling tool includes data from about 5143 breast cancer patients examining several prognostic parameters, including overall survival (OS), relapse-free survival (RFS), and distant metastasis-free survival (DMFS). Our finding (Figure 5A) demonstrated a strong association between the examined genes’ signature and the breast cancer basal subtype, so we focused our analysis on this subtype. As presented in Figure 8A and B, breast cancer patients with elevated expression of PTGS2/ESR2/EGFR/JUN/MMP2 genes’ signature displayed significantly poor OS (*P* =  0.026) and DMFS (*P* =  0.0066) and consequently unfavorable prognosis. However, we didn’t find a significant correlation between high genes’ signature expression and the RFS parameter (*P* = .25). This data should shed light on the synergetic oncogenic role of the concurrent expression of PTGS2/ESR2/EGFR/JUN/MMP2 genes’ signature in breast cancer patients. Thus, expression is coupled with higher malignancy degrees and unfavorable patients’ prognoses.

### PTGS2/ESR2/EGFR/JUN/MMP2 Genes’ Signature as a Prognostic Marker in Other Types of Cancers

**Figure 9.**
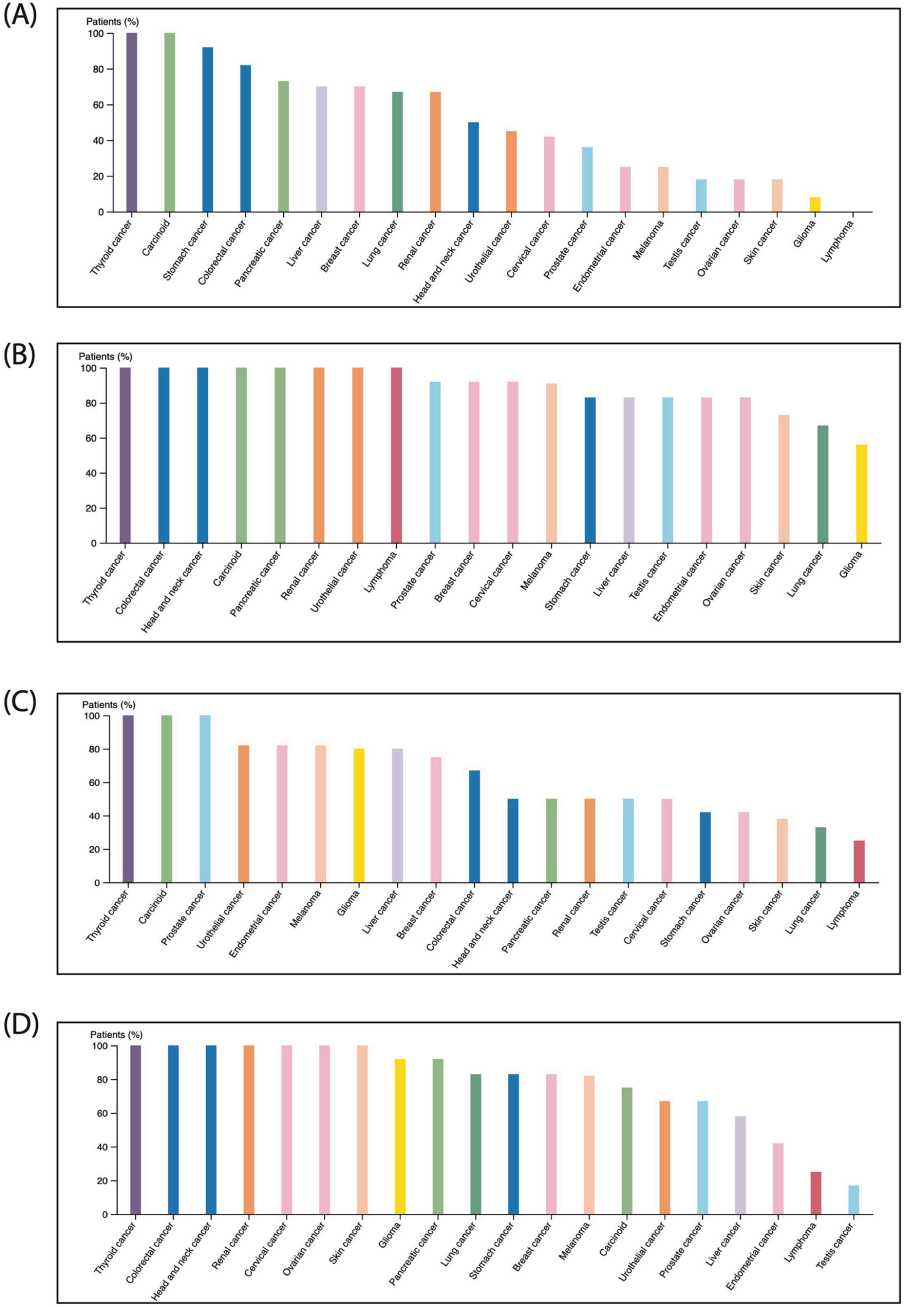
Examination of PTGS2/ EGFR/JUN/and MMP2 protein level among various human cancers: Protein expression profiles of (A) PTGS2, (B) EGFR, (C) MMP2, and (D) JUN demonstrated in twenty different human cancers, the data generated using THE HUMAN PROTEIN ATLAS online tool.

**Figure 10.**
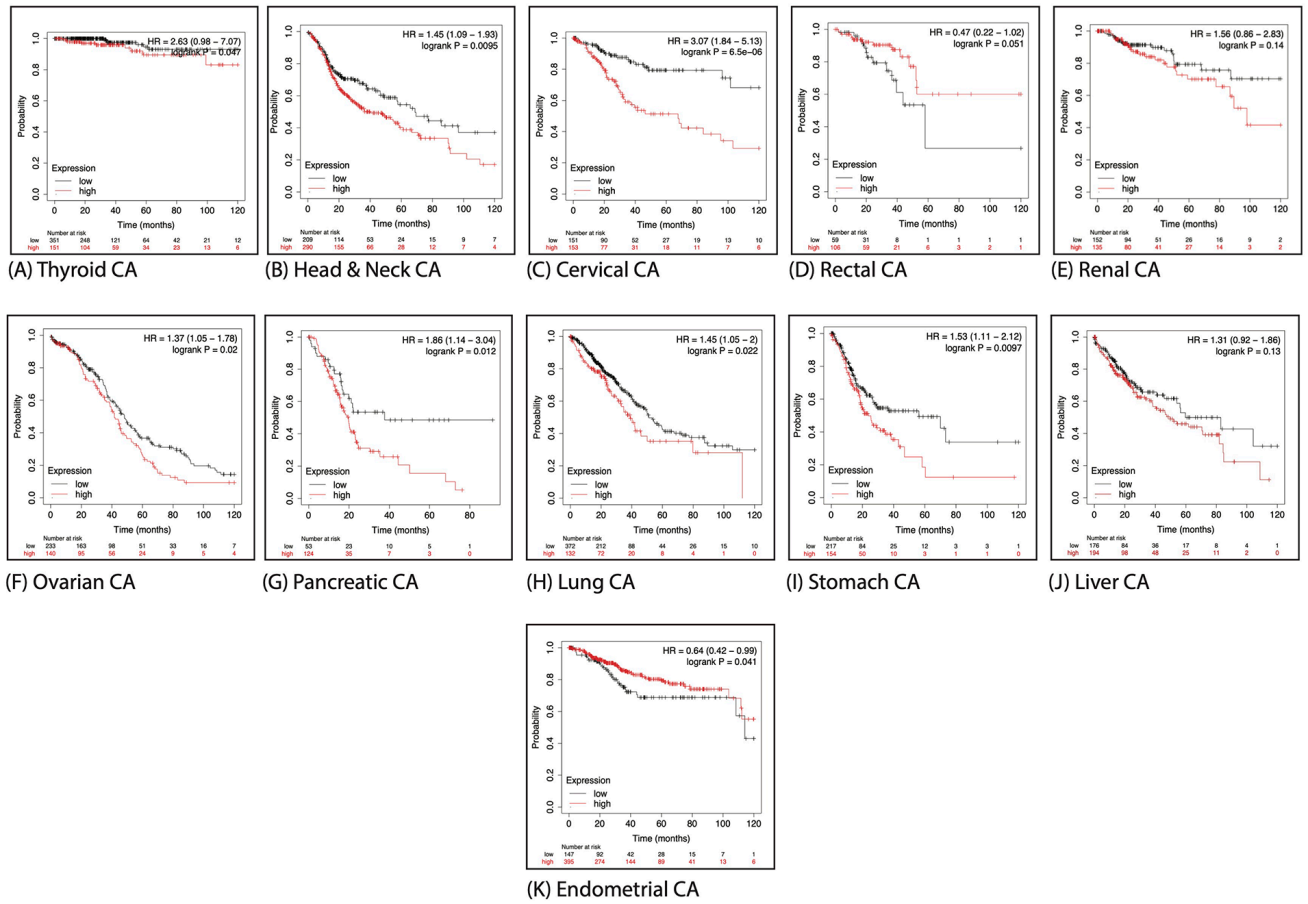
Prognostic value of the PTGS2/ESR2/EGFR/JUN/and MMP2 genes’ signature expression in other human cancers types: (A), (B), (C), (F), (G), (H), and (I) High expression of PTGS2/ESR2/EGFR/JUN/and MMP2 genes’ signature is associated significantly with poor overall survival (OS) in thyroid cancer (*P* = .047), head and neck cancer (*P* = .0095), cervical cancer (*P* = .6.5^^−06^), ovarian cancer (*P* = .02), pancreatic cancer (*P* = .012), lung cancer (*P* = .022), and stomach cancer (*P* = .0097). (D), (E), and (J) No significant association between the high expression level of these genes’ signature and OS of rectal, renal, and liver cancer patients (*P* = .05, *P* = .14, and *P* = .13, respectively). (K) Marginally significant (*P* = .041) correlation between high expression of this genes’ signature and better OS in patients with endometrial carcinomas. All curves were obtained using the KM-Plotter analysis prognostic analysis database.

Myrrh extracts/metabolites were described to produce cytotoxic effects on diverse types of human cancers^18-25^; next, we explored the contribution of the current genes’ signature in the pathology of other cancer types. The HUMAN PROTEIN ATLAS online tool was employed to study the expression of PTGS2/ESR2/EGFR/JUN/MMP2 genes’ signature components at protein levels in different types of human cancers, as seen in Figure 9A to D, immunohistochemistry analysis of 20 other human cancers showed variant protein expression levels of each component individually, with the highest protein level found in thyroid, colorectal, and carcinoid cancers. In contrast, the lowest levels were detected in gliomas, lymphoma, and testicular cancers. Next, an online Kaplan-Meier plot was used to assess the predictive value of the PTGS2/ESR2/EGFR/JUN/MMP2 genes’ signature expression level on OS in numerous human cancers. As shown in Figure 10A to K, our analysis demonstrated a significant association between low expression of the examined genes’ signature and prolonged OS in the following patients with thyroid cancer (*P* =  .047), head and neck cancer (*P* =  .0095), cervical cancer (*P* = .6.5^^−06^), ovarian cancer (*P* = .02), pancreatic cancer (*P* =  .012), lung cancer (*P* =  .022), and stomach cancer (*P* =  .0097). We also found a marginally significant (*P* = .041) correlation between high expression of this genes’ signature and better OS in patients with endometrial carcinomas. On the other hand, we didn’t find a significant association between these genes’ signature expression level and OS of rectal, renal, and liver cancer patients (*P* =  .05, *P* = .14, and *P* = .13, respectively). Altogether, these findings further authenticate that the oncogenic contributions of these genes could extend to involve other human cancers in addition to breast cancer tumorigenesis. Thus, patients’ cancers exhibited high expression levels of PTGS2/ESR2/EGFR/JUN/MMP2 genes’ signatures are associated with poor prognosis.

## Discussion

Natural products interact with a wide range of targets, which makes them more favorable than conventional chemotherapy. Furthermore, natural compounds have become increasingly popular as anticancer treatments because of their significant therapeutic potential and low systemic toxicity.^51^ Myrrh (the resin from *Commiphora myrrha* Engl.) extract is a well-known natural medicinal plant with substantial therapeutic properties due to the action of its many active metabolites.^20,26^ Given that most therapeutic drugs have serious side effects that lower quality of life, myrrh offers a potential, less harmful therapeutic option and shows promise against cancer and inflammatory illnesses.^25^ Enormous studies have investigated the anti-cancer effects of myrrh and its metabolites in many cancers, including breast cancers.^18-25^ Cancer drugs that target multiple pathways involved in apoptosis, a crucial feature of cancer cells, are among the most effective non-surgical treatments for different types of cancer. In this respect, several studies have reported using myrrh components in apoptotic induction and blockage of tumor cell proliferation.^26,52,53^ Myrrh extract has also shown anti-proliferative properties in different human cancer cells, including prostate cancer and leukemia,^54^ by inhibiting the growth and division of prostate cancer cells, consequently slowing the progression of the disease.^55^ Similarly, Commiphora myrrha components were found to show anti-proliferative activity in glioblastoma by activating the p38MAK pathway.^56^ Moreover, the bioactive ingredient of myrrh (bisabolene) showed a significant inhibitory effect on breast cancer cellular growth.^31^

In the present study, we identified numerous myrrh-active molecules that were predicted, in silico, to target many breast cancer genes, proposing their potential contribution to breast tumor evolution. In alignment with our findings, previous reports, using several in silico database analyses, have predicted the possible molecular mechanisms by which frankincense and myrrh, the main ingredients of the *Xihuang* pill, can inhibit hepatic cellular carcinomas^57^ and breast tumor growth, invasion, angiogenesis, and regulating cancer stemness capacity.^18^ Furthermore, our in silico TCMSP investigations showed that Guggulsterone (GUG) is one of the myrrh bioactive components that target breast cancer genes (i.e. PGR and PTGS2). Indeed, GUG was described as the most potent bioactive component of myrrh, which increases the response of the multidrug-resistant MCF-7/DOX cells to the doxorubicin (DOX) chemotherapy by suppressing the expression of Bcl-2 and P-glycoprotein.^21^ Additionally, *in vitro* and *in vivo* reports and network pharmacology platforms have identified Z-Guggulsterone (Z-GS), an active myrrh component, as an encouraging therapeutic agent in treating triple-negative breast cancer.^33^ The ethanolic extraction of the Arabic *Commiphora myrrha* resin produced several vital compounds: CM1 (2-methoxyfuranodiene), CM2 (2-acetoxyfuranodiene), and β-elemene.^26,30^ Our TCMSP results also found that PTGS2 (the key targeted gene) is affected by the CM1 and β-elemene components. Indeed, the *in vitro*examination reported a potent cytotoxic, antiproliferative, apoptosis induction and cell cycle arrest delivered by these compounds (CM1, CM2, and β-elemene) on the MCF-7 breast cancer cells^30^ and glioblastomas.^26^ Similarly, another in silico-based study has identified the secondary myrrh constituents that were computationally predicted to exhibit anticancer, anti-inflammatory, and wound-healing properties.^58^ Nevertheless, the exact molecular mechanisms of the antitumor proprieties of myrrh-active ingredients in breast cancer have not been fully explored. Therefore, using in silico exploration in the current work allowed us to examine myrrh-specific ingredients predicted to target several genes and their signaling pathways in breast cancer. Additionally, gathering the power of distinct bioinformatics platforms resulted in constructing a genes’ signature consisting of five positively correlated genes identifying as PTGS2/ESR2/EGFR/JUN/MMP2 signature. The examined gene signature components (individually or as a Merged Gene Set) demonstrated a positive correlation with advanced breast cancer subtypes at mRNA expression and protein levels, suggesting their participation during cancer progression. Notably, the concurrent expression of this genes’ signature is linked negatively with breast cancer patients’ prognosis.

Our study recognized PTGS2 as a master targeted gene by most of myrrh active molecules. Prostaglandin-endoperoxide synthase 2 (*PTGS2*) is an enzyme that encodes the cyclooxygenase-2 (COX-2) enzyme. This rate-limiting enzyme facilitates the production of prostaglandins from the arachidonic acid, hence playing an essential role in inflammation.^59^ Numerous studies have reported the pivotal involvement of PTGS2/COX-2 in tumor growth, invasion, and metastases of many cancers, including lung, colon, stomach, urinary, and breast cancers.^59-63^ Moreover, PTGS2 was reported to participate in several oncogenic signaling pathways related to cell proliferation, growth, survival, angiogenesis, inflammation, metastasis, and mutagens production^61,63,64^ through activation of the crosstalk between SP/NK1R and COX-2 pathways^61^ and regulation of TGFβ/Smad3-dependent pathway. Indeed, blocking COX-2 activity using a potent, selective pharmacological inhibitor, celecoxib inhibited TGFβ mediated tumorigenesis and enrichment of the primary breast cancer stem cells, CD24^low^CD44^high^ and ALDH+ populations.^62^ In agreement with our PPI network and gene expression profiles findings, the gene expression and functional analyses of the disseminated breast cancer cells identified the PTGS2 and EGFR ligands as mediators of extravasation that enhance the invasion of breast cancer cells into the lung and the brain tissues through crossing the blood-brain barriers.^65^

Furthermore, PTGS2 protein revealed a high expression profile in aggressive breast cancers, including the basal and TNBC subtypes, highlighting its contribution during breast cancer advancement and progression.^59,61,62^ Also, in alignment with our IHC findings, elevated COX-2 expression was detected in a high proportion of breast cancer samples^66^ and revealed a strong association with aggressive tumor biology characterized by large size, higher grade, advanced stage, lymph node involvement,^67,68^ ER and PR negative, and HER-2 positivity^63,66,69^ observed explicitly in postmenopausal patients over the age of 50.^63^ Thus, breast cancer patients with upregulation of PTGS2 level display poor survival outcomes and prognosis, similar to our data, where we found that the expression of PTGS2/ESR2/EGFR/JUN/MMP2 genes’ signature is correlated with unfavorable patient prognosis in different human cancers, including breast cancer, especially in the basal subtype.^62,69^ A meta-analysis of twenty-one studies involving around 6739 breast cancer patients demonstrated that high expression level of COX-2 is significantly associated with short disease-free survival, short overall survival, and poor prognosis.^67^ Collectively, these findings indicate the importance of targeting PTGS2 (COX-2) expression and activity in lessening the progression of breast cancer.

*Different innovative strategies have been developed and implanted to inhibit COX-2 activity and prevent breast cancer development*.^70,71^ Plenty of anti-inflammatory drugs, including nonsteroidal anti-inflammatory drugs (NSAIDs) that have been established primarily to treat inflammatory illnesses, were also found to deliver chemoprevention effects for several cancers, including breast cancers.^70^ These molecules act by inhibiting COX-2 activity and the subsequent production of prostaglandin, suppressing the oncogenic nuclear factor-κB (NF-kB) signaling pathway and preventing inflammation-induced angiogenesis.^70^ Additionally, several dietary phytochemicals exhibited anti-oxidant and anti-inflammatory properties, which regulate cell proliferative pathways and apoptosis signaling, thus reducing or hindering breast cancer occurrence.^70^ *Other pharmacological substances include* Licofelone (dual COX and lipoxygenase (LOX) inhibitor) and diaryl heterocycle (pyrazole scaffolds compound), which *were reported to produce both anticancer (breast, liver, and colon cancers) and COX-2 suppressing effects*.^71^ Besides mono-therapy, the introduction of a combination regimen consisting of NK1R antagonists (a receptor for neuropeptide Substance P) and COX-2 inhibitors demonstrated a significant reduction in breast cancer stemness capacity in TNBC cells thus improving the treatment sensitivity and lessening the risk of recurrence and relapse.^6^ These data strongly suggested that the introduction of myrrh natural medicine, via targeting the PTGS2, would provide a potent remedy in treating breast cancer patients.

Our PPI network further indicated the functional and/or physical interaction of our genes’ signature components with other partner proteins in breast cancer pathogenesis. Moreover, leveraging the KEGG enrichment analysis tool, we appreciated the contribution of the examined genes’ signature in different cancer signaling pathways, including breast cancers. Thus, by targeting these genes’ signatures, myrrh can regulate multiple molecular signaling pathways and metabolic processes involved in carcinogenesis. Within this context, a previous report using GO and KEGG pathway enrichment analyses reported the potential favorable implication of myrrh in TNBC treatment via downregulation of several cancer pathways, cellular proliferation, protein bindings, apoptotic mechanism, proteoglycans in cancer, and PI3K-Akt signaling.^33^ In alignment with published literature, our search concluded the promising engagement of myrrh in treating various cancer types by targeting these genes’ signatures. Zheng et al^57^ reported that treated U266 multiple myeloma cells with myrrh ethanol extracts inhibited cellular proliferation by regulating JAK/STAT signaling pathways. Myrrh ethanol extracts affect various metabolic profiles, including amino acid, glucose, vitamin, and lipid metabolism.^72^ Similarly, through regulation of the EGFR-mediated PI3K/Akt and MAPK signaling pathways activity, the combination of frankincense and myrrh was found to significantly inhibit angiogenesis and suppress the growth of hepatocellular carcinoma in nude mice.^57^ It also inhibited the proliferation of the human promyelocytic leukemia cell line HL-60 by blocking the receptor tyrosine kinase activity in cell lines and mice models.^73^ In the context of gastric cell carcinoma, different biological studies confirmed the tumor suppression effects of myrrh on cellular growth, invasion, and apoptosis in vitro and in animal models. These effects were delivered by downregulating the expression of PCNA, COX-2 (PTGS2), and Bcl-2 and enhancing the expression of Bax proteins in the cancer cells.^74^

Fruitful *in vitro* and *in vivo* studies have investigated the anti-tumorigenic role exhibited by myrrh in breast cancers.^16,17,19,74-76^ Ellagic acid (EA), a potent polyphenol compound of myrrh, was found to induce tumor cell apoptosis and autophagy and suppress cellular proliferation, invasion, and metastasis in different cancer types such as gastric cancer, liver cancer, pancreatic cancer, breast cancer, colorectal cancer, lung cancer. These tumor inhibitor effects are attributed to the capacity of EA to mediate and regulate many molecular pathways involving the VEGFR-2 signaling pathway, Notch signaling pathway, PI3K/Akt signaling pathway, Bcl-2 / Bax signaling pathway, TGF-β/Smad3 signaling pathway, and COX-2 signaling pathway.^75^ Likewise, Guggulsterone (an active myrrh ingredient) can block breast tumor initiation, promotion, and metastasis through modulation of cell cycle proteins (cyclin D1 and c-Myc), downregulation of anti-apoptotic genes (Bcl-2 and survivin) and Akt activity, activation of caspases, and inhibition of metastasis mediated genes (MMP-9, COX-2, and VEGF).^19,76^ Also, XH pills that contain myrrh have shown promising results in treating breast neoplasms^22^ and improved immunity and a 2-year survival rate in the treating group.^23^ Moreover, XH showed efficacy in treating benign breast lesions and hyperplasia and precluding malignant transformation.^18,24^ Besides myrrh’s role in cancer, myrrh’s anti-inflammatory effects extend to treating cerebral ischemic injuries^16^ and knee osteoarthritis.^17^ This outcome was obtained by downregulation of the expression of the pro-inflammatory factors *COX-2, IL-6, IL-1β, and TNF-α* in rat models of brain ischemic injury^16^ and regulation of the expression of the transcription factor JUN, IL-6, IL-1β, and Mitogen-activated protein kinase 1 (MAPK1) in the knee osteoarthritis.^17^ Jointly, myrrh-active compounds deliver multifaceted biological effects, designating myrrh as an attractive therapeutic tool in diverse illnesses, particularly cancers.

## Conclusion

The findings of the present work acknowledged the valuable anti-cancer and anti-inflammatory activity of myrrh; this study predicted that specific myrrh-bioactive components exhibited antitumorigenic effects in breast cancer by targeting distinctive genes that mediate breast cancer pathogenesis. It also suggested that myrrh could regulate numerous molecular pathways during breast cancer development. This manuscript uses various bioinformatics datasets to evaluate myrrh’s role and designate a distinctive gene signature. To the best of our knowledge, this is the first in silico analysis in myrrh research that should shed light on introducing myrrh as an anti-cancer therapeutic candidate. Subsequent pre-clinical investigations of its pharmacological activity and precise mechanisms of action should be conducted to validate its anticancer effects further. This study could be used as a starting point to explore experimentally the potential biological properties of each active compound.

**Table:** 

Abbreviation	Full Term
ALDH+	Aldehyde dehydrogenase 1
bc-GenExMiner v5.0	The Breast Cancer Gene-Expression Miner v5.0
Bcl-2	B-cell lymphoma 2
CCN2	Cellular Communication Network Factor 2
CCNB1	Cyclin B1
CD24	Cluster of differentiation 24
CD44	Cluster of differentiation 44
CM1	2-methoxyfuranodiene
CM2	2-acetoxyfuranodiene
c-Myc	Cellular myelocytomatosis oncogene
COX-2	Cyclooxygenase-2
CTSD	Cathepsin D
CXCL3	C-X-C Motif Chemokine Ligand 3
DMFS	Distant Metastases-Free Survival
DOX	Doxorubicin
EA	Ellagic acid
EGFR	Epidermal growth factor receptor
ER	Estrogen receptors
EREG	Epiregulin
ERK1	Extracellular signal-regulated kinase 1
ESR1	Estrogen receptor 1/alpha
ESR2	Estrogen receptor 2/beta
G2/M	Second growth phase/mitosis
GO	Gene Ontology
GOBO	Gene expression-based Outcome for Breast Cancer
GUG	Guggulsterone
HBEGF	Heparin binding EGF like growth factor
HER2	Human epidermal growth factor receptor2
HR	Hormonal Receptors
HSP90AB1	Heat shock protein 90 alpha family class B member 1
IHC	Immunohistochemistry
IL-1β	Interleukin-1 beta
IL-6	Interleukin 6
JAK/STAT	The Janus kinase/signal transducers and activators of transcription
JUN	Transcription factor AP-1
KEGG	Kyoto Encyclopedia of Genes and Genomes
KM-plot	Kaplan-Meier plots
LOX	Lipoxygenase
MAPK	Mitogen-activated protein kinase
MAPK1	Mitogen-activated protein kinase 1
MMP1	Matrix Metallopeptidase 1
MMP14	Matrix Metallopeptidase 14
MMP2	72 kDa type IV collagenase
MMP-9	Matrix Metallopeptidase 9
mRNA	Messenger RNA
NF-κB	Nuclear factor-κB
NK1R	Neurokinin 1 receptor
NOCA1	Nuclear receptor coactivator 1
NOCA3	Nuclear receptor coactivator 3
NSAIDs	Nonsteroidal anti-inflammatory drugs
OS	Overall Survival
p53	Protein 53
PGR	Progesterone receptors
PI3K-Akt	Phosphatidylinositol 3-kinase/protein kinase B
PLK1	Polo like kinase 1
PPI	Protein-protein interaction
PTGS2	Prostaglandin-endoperoxide synthase
RFS	Relapse-Free Survival
SD	Standard deviation
Smad3	Mothers against decapentaplegic homolog 3
SP1	Specificity Protein 1
TCMSP	Traditional Chinese Medicine Systems Pharmacology Database and Analysis Platform
TGFβ	Transforming growth factor β
TIMP4	Metallopeptidase Inhibitor 4
TNBC	Triple-negative breast cancer
TNF	Tumor necrosis factor
TNF-α	Tumor necrosis factor alpha
VEGFR	Vascular endothelial growth factor receptor
XH	Xihuang pill
Z-GS	Z-Guggulsterone
